# Longitudinal Collection of Patient-Reported Outcomes and Activity Data during CAR-T Therapy: Feasibility, Acceptability, and Data Visualization

**DOI:** 10.3390/cancers14112742

**Published:** 2022-05-31

**Authors:** Laura B. Oswald, Xiaoyin Li, Rodrigo Carvajal, Aasha I. Hoogland, Lisa M. Gudenkauf, Doris K. Hansen, Melissa Alsina, Frederick L. Locke, Yvelise Rodriguez, Nathaly Irizarry-Arroyo, Edmondo J. Robinson, Heather S. L. Jim, Brian D. Gonzalez, Kedar Kirtane

**Affiliations:** 1 Department of Health Outcomes and Behavior, Moffitt Cancer Center, 12902 USF Magnolia Dive, MFC-HOB, Tampa, FL 33612, USA; shelly.li@moffitt.org (X.L.); aasha.hoogland@moffitt.org (A.I.H.); lisa.gudenkauf@moffitt.org (L.M.G.); yvelise.rodriguez@moffitt.org (Y.R.); nathaly.irizarry-arroyo@moffitt.org (N.I.-A.); heather.jim@moffitt.org (H.S.L.J.); brian.gonzalez@moffitt.org (B.D.G.); 2 Department of Biostatistics and Bioinformatics, Moffitt Cancer Center, Tampa, FL 33612, USA; rodrigo.carvajal@moffitt.org; 3 Department of Blood and Marrow Transplant and Cellular Immunotherapy, Moffitt Cancer Center, Tampa, FL 33612, USA; doris.hansen@moffitt.org (D.K.H.); melissa.alsina@moffitt.org (M.A.); frederick.locke@moffitt.org (F.L.L.); 4 Center for Digital Health, Moffitt Cancer Center, Tampa, FL 33612, USA; edmondo.robinson@moffitt.org; 5 Department of Head and Neck-Endocrine Oncology, Moffitt Cancer Center, Tampa, FL 33612, USA; kedar.kirtane@moffitt.org

**Keywords:** feasibility studies, hematologic neoplasms, immunotherapy, adoptive, patient-reported outcome measures, wearable electronic devices

## Abstract

**Simple Summary:**

Patients treated with chimeric antigen receptor T-cell therapy (CAR-T) are at risk for severe toxicities post-treatment. Patient-reported outcomes and activity data from fitness trackers could be helpful for monitoring patients after CAR-T. The aim of this pilot study was to test the feasibility and acceptability of having patients complete surveys on a rigorous schedule and wear a Fitbit tracker continuously, from before their CAR-T infusion through 90-days post-infusion. In a sample of 12 patients with hematologic malignancies, we demonstrated feasibility and acceptability, with high rates of behavioral adherence to the study procedures. These findings suggest that large-scale data collection efforts using these procedures will be successful. In turn, patient-reported outcomes and activity data could be used to identify predictors of severe CAR-T-related toxicities.

**Abstract:**

Background: Clinicians must closely monitor patients for toxicities after chimeric antigen receptor T-cell therapy (CAR-T). Patient-reported outcomes (PROs) (e.g., toxicities, quality of life) and activity data (e.g., steps, sleep) may complement clinicians’ observations. This study tested the feasibility and acceptability of collecting PROs and activity data from patients with hematologic malignancies during CAR-T and explored preliminary data patterns. Methods: Participants wore a Fitbit tracker and completed PROs at several timepoints through 90-days post-infusion. Feasibility was assessed with a priori benchmarks for recruitment (≥50%), retention (≥70%), PRO completion (≥70%), and days wearing the Fitbit (≥50%). Acceptability was assessed with participant satisfaction (a priori benchmark > 2 on a 0–4 scale). Results: Participants (N = 12) were M = 66 years old (SD = 7). Rates of recruitment (68%), retention (83%), PRO completion (85%), and days wearing the Fitbit (85%) indicated feasibility. Satisfaction with completing the PROs (M = 3.2, SD = 0.5) and wearing the Fitbit (M = 2.9, SD = 0.5) indicated acceptability. Preliminary data patterns suggested that participants with better treatment response (vs. progressive disease) had a higher toxicity burden. Conclusions: Longitudinal PRO and activity data collection was feasible and acceptable. Data collected on a larger scale may be used to specify risk prediction models to identify predictors of severe CAR-T-related toxicities and inform early interventions.

## 1. Introduction

Chimeric antigen receptor T-cell therapy (CAR-T) has revolutionized treatment for patients with hematologic malignancies [1,2,3,4,5,6,7,8], with a recent meta-analysis finding that CD19 CAR-T produced a complete response in more than 50% of patients across 27 studies [9]. Yet despite the potential benefits, a large proportion of patients experience severe CAR-T-related toxicities that can be dangerous if not detected and treated early. For example, cytokine-release syndrome (CRS) is a potentially life-threatening constellation of toxicities including fever, tachycardia, hypotension, and hypoxemia [10,11,12]. Another toxicity, immune effector cell-associated neurotoxicity syndrome (ICANS), often presents after CRS as confusion, encephalopathy, and in severe cases, seizures [12].

Due to the risk of severe toxicities, clinicians must closely monitor patients after CAR-T infusion. However, clinicians can under-report the incidence and severity of toxicities by 50% or more relative to patient reports [13,14]. Thus, patient-reported outcomes (PROs) are a critical complement to clinician observations. In a seminal trial, Basch and colleagues found that systematically monitoring PROs among patients with advanced solid malignancies during chemotherapy was associated with longer survival, fewer hospitalizations, longer treatment continuation, better health-related quality of life (HRQOL), and was cost-effective relative to usual care [15,16,17]. Benefits were thought to result from early detection and treatment of toxicities. Given the potential for severe toxicities after CAR-T, systematically monitoring PROs could generate valuable data to inform clinical care and improve patient outcomes.

In addition, wearable devices such as fitness trackers are increasingly used to passively collect dense activity data from patients (e.g., steps, sleep) [18,19], and emerging studies suggest that activity data may provide insights into patients’ health above and beyond PROs alone. For example, a pilot study used wearables to collect activity data and smartphones to collect PROs from ten patients with advanced gynecologic malignancies receiving palliative chemotherapy [20]. Using activity data, investigators identified two patients with poor health status before PROs indicated severe toxicities. Thus, collecting activity data via wearables may have additional value to systematically monitoring PROs during cancer treatment.

The goal of this study was to apply these procedures and test the feasibility and acceptability of collecting longitudinal PROs and activity data from patients with hematologic malignancies receiving CAR-T. This study also explored preliminary patterns of PROs and activity data with clinical and laboratory outcomes.

## 2. Materials and Methods

### 2.1. Participants

Study activities were approved by Advarra Institutional Review Board (Pro00046848). Participants were recruited from an NCI-designated comprehensive cancer center as part of a larger study with the following eligibility criteria: (1) ≥18 years old; (2) scheduled to receive any adoptive cellular immunotherapy (ACT) for any cancer diagnosis; (3) could speak and read English; (4) without documented or observable psychiatric or neurologic diagnoses that could preclude participation; (5) able to complete electronic surveys; and (6) able to provide informed consent. In the larger study, an a priori recruitment goal was set as N = 15 based on guidelines for appropriate sample sizes to estimate feasibility and acceptability in single-arm pilot studies [21]. These analyses focused on a subset of the study participants who specifically had hematologic malignancies (e.g., multiple myeloma, lymphoma) and had received CAR-T therapies.

### 2.2. Recruitment, Data Collection, and Measures

From March–June 2021, a trained research coordinator worked with clinic staff to identify potentially eligible patients, screened patients’ electronic medical records (EMRs), and approached patients to introduce the study, confirm eligibility, allow time for questions, and solicit informed consent verbally. Consented participants were loaned a Fitbit Inspire 2 fitness tracker, an iPad with cellular connectivity, and associated device chargers. They were instructed to wear the Fitbit on their non-dominant wrist for 91 days beginning the day of CAR-T infusion (day 0). The Fitbit and iPad were configured to automatically sync and upload the activity data to Fitbit cloud servers, and in-house tools were developed to retrieve the data for local storage. Participants were also instructed to complete PRO surveys using REDCap, a secure and HIPAA-compliant internet-based data capture tool [22]. Links to PRO surveys were sent to participants via text message using Twilio (San Francisco, CA, USA), an SMS text message software integrated with REDCap, and via email. There were 14 PRO assessment timepoints informed by recommendations for monitoring PROs after CAR-T [23]: enrollment (baseline), day of infusion (day 0), daily for one week (days 1–7), weekly for one month (days 14, 21, and 30), and monthly for three months (days 60 and 90). The contents of each PRO assessment are shown in Table 1. Participants were compensated USD 10 for completing each of the PRO assessments and another USD 60 if they wore the Fitbit for at least 50% of the study days (maximum incentive USD 200/participant). Study staff were available to assist with technical problems, as needed.

Demographic and clinical characteristics. Participants self-reported their demographics (e.g., date of birth, gender, race/ethnicity, education, income) and medical comorbidities using the Charlson Comorbidity Index (CCI) [24]. Clinical data were confirmed via EMR review and additional clinical data were abstracted, including laboratory values for C-reactive protein (CRP) and ferritin between days 0–30 (known predictors of CRS) [25], hospitalizations between days 0–30, and treatment response at day 90. Treatment response was defined as a partial response to treatment or better vs. progressive disease. Progressive disease was indicated in cases of persistent disease in the bone marrow, worsening disease seen on PET/CT imaging as measured by either avidity or new lesions, or disease-specific indications (e.g., rise in myeloma serum or urine markers by >25% for patients with multiple myeloma).

Feasibility and acceptability. Feasibility and acceptability were assessed using a priori benchmarks consistent with published guidelines [26] and prior studies using similar protocols [20,27]. Study procedures were considered feasible if ≥50% of eligible patients consented (recruitment), ≥70% of participants completed the day 90 PRO assessment (retention), ≥70% of PRO assessments were completed (adherence to PRO protocol), and participants wore the Fitbits for ≥50% of study days (adherence to Fitbit protocol). At day 30, acceptability was assessed with a study-specific survey developed by the study team based on experience using similar measures [28,29]. Participants rated their agreement with positive statements about completing the PRO surveys (7 items) and wearing the Fitbit (5 items) on a Likert-type scale from 0 (strongly disagree) to 4 (strongly agree). Item responses within each domain were averaged, and higher scores indicated better acceptability. Study procedures were considered acceptable if average scores were >2 (i.e., better than neutral).

HRQOL. The 27-item Functional Assessment of Cancer Therapy-General (FACT-G) assessed total HRQOL and four well-being domains (i.e., physical, social, emotional, and functional) [30]. Participants rated the degree to which items applied to them on a Likert-type scale from 0 (*not at all*) to 4 (*very much*). Items were summed, with higher scores indicating better HRQOL. Average total HRQOL ≤ 70, physical well-being ≤ 18, social well-being ≤ 19, emotional well-being ≤ 15, and functional well-being ≤ 14 indicated clinically low HRQOL [31]. The 31-item and NIH-developed PRO Measurement Information System (PROMIS)-29+2 Profile v2.1 assessed depression, anxiety, fatigue, pain interference, physical function, sleep disturbance, ability to participate in social roles and activities, cognitive function, and global pain [32,33]. Participants rated their pain from 0–10, with higher scores indicating worse pain. For all other PROMIS scales, participants rated the degree to which items applied to them on Likert-type scales from 0 to 4, and standardized T-scores were calculated (normative M = 50, SD = 10). Higher PROMIS scores indicate more of the construct measured. Thus, lower scores were better for negative constructs (i.e., depression, anxiety, fatigue, pain interference, sleep disturbance) (scores 55–59 mild, 60–69 moderate, ≥70 severe) and higher scores were better for positive constructs (i.e., physical function, ability to participate in social roles and activities, cognitive function) (scores ≤ 30 severe, 31–40 moderate, 41–45 mild). The 7-item FACT-G7 assessed top-priority concerns previously identified by heterogeneous cancer patients [34]. Participants rated the degree to which items applied to them on a Likert-type scale from 0 (not at all) to 4 (very much). Items were summed, with higher scores indicating better HRQOL. Average scores ≤ 16 indicated clinically low HRQOL [31].

Toxicity burden. The NCI-developed PRO version of the Common Terminology Criteria for Adverse Events (PRO-CTCAE) is a library of 124 items that assess the frequency, severity, and/or interference with usual or daily activities of 78 toxicities [35,36]. It was designed so investigators can select individual items relevant to a given diagnosis and/or treatment. This study assessed 31 toxicities with 47 items based on consensus among study team experts about relevance for patients undergoing ACT (e.g., decreased appetite, nausea, vomiting, constipation, diarrhea, shortness of breath). Participants rated a toxicity’s frequency (never to almost constantly), severity (none to very severe), and interference with their usual or daily activities (not at all to very much) on Likert-type scales from 0–4. Within-person composite grades were calculated for each toxicity using all available data (i.e., frequency, severity, and/or interference) [37]. PRO-CTCAE composite grades map onto clinician-rated CTCAE grades, where 0 indicates that a toxicity is absent, 1 is mild, 2 is moderate, 3 is severe, 4 is life-threatening, and 5 is toxicity-related death. PRO-CTCAE composite grades were capped at 3 (severe) [37] and used to calculate a summary of toxicity burden according to the following equation [38,39]:

$$Toxicity\;Index=\overset{n}{\underset{i=1}{\sum }}\frac{X_{i}}{\prod_{j=1}^{i-1}\left(1+X_{j}\right)}$$

Toxicities were ranked in descending order of severity, assigned decreasing weights, and summed. This approach accommodates the differential impact of multiple toxicities and yields an easily interpretable score, where the number before the decimal indicates the highest grade reported by a participant (i.e., a participant reporting only one grade 3 toxicity would have a TI of 3.0), and the numbers after the decimal indicate other toxicities beyond the highest grade, with lower grade toxicities contributing less to the final score. For example, a participant reporting one grade 3 toxicity and two grade 2 toxicities would have a TI of 3.67, whereas a participant reporting two grade 3 toxicities would have a TI of 3.75, indicating a worse toxicity burden.

Activity data. Participants’ daily activity (i.e., steps) and sleep efficiency (i.e., percent of time in bed spent asleep) were continuously collected via Fitbit trackers for 91 days, from the day of CAR-T infusion (day 0) through day 90 post-infusion. Sleep efficiency scores <85% were considered clinically low [40].

### 2.3. Statistical Analyses

Analyses were conducted with SAS version 9.4 (SAS Institute, Cary, NC, USA) and R version 4.1. Summary statistics were used to describe the sample characteristics. Frequencies and percentages were calculated for feasibility. Means and standard deviations were calculated for acceptability and PROs. At a given timepoint, steps and sleep efficiency were averaged across the preceding timeframe. For example, a participant’s average steps at day 14 were the average of their steps across days 8–14. Preliminary patterns of PROs and activity were visually explored with clinical and laboratory outcomes in two ways. First, participants were categorized as having a partial treatment response or better vs. progressive disease at day 90, and the average TI was plotted over time by group. Second, two example participants were selected: one who achieved a partial response or better at day 90 and one with progressive disease. Their individual data from several sources were graphed together to visualize the depth of information achieved from the combination of PRO, activity, and clinical data.

## 3. Results

### 3.1. Sample Characteristics

Table 2 shows the sample characteristics (N = 12). Participants were an average of 66 years old (SD = 7), and half were female (50%). Most identified as White (83%) and non-Hispanic (92%). More than half were diagnosed with multiple myeloma (58%). Per institutional protocols, all patients received CAR-T as inpatients, and the average hospital stay was 10 days (SD = 7).

### 3.2. Feasibility and Acceptability

**Figure 1:**
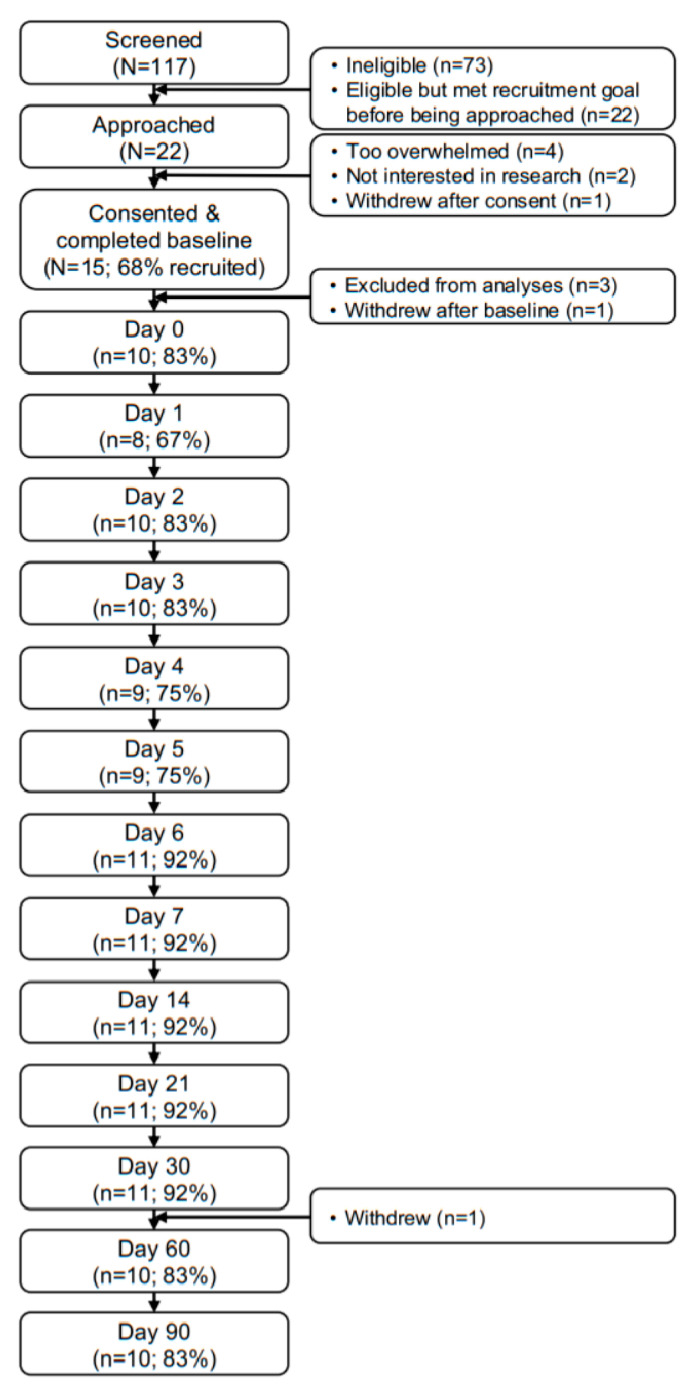
Participant flow diagram.

Figure 1 shows the participant flow through the study. All a priori feasibility and acceptability benchmarks were met. In total, 22 eligible patients were approached for the larger study and 15 (68%) consented to participate. This paper reports on the 12 participants with hematologic malignancies who received CAR-T. In total, 10 of the 12 analyzed participants (83%) were retained through day 90. Collectively, participants completed 85% of all PRO assessments (143 PRO assessments completed of 168 administered), and they wore the Fitbits for 85% of study days (928 days worn of 1092 total study days). Average satisfaction with the PRO assessments was 3.2 (SD = 0.5), indicating participants agreed to strongly agreed with positive statements about the PRO assessments. Average satisfaction with wearing the Fitbits was 2.9 (SD = 0.5), indicating participants were neutral to agreed with positive statements about wearing the Fitbits. 

### 3.3. Preliminary Patterns of PROs and Activity Data

Table 3 shows average HRQOL scores, TI, steps, and sleep efficiency at each timepoint.

HRQOL. Based on validated cutoffs, the average FACT-G total HRQOL scores were within the normal range at all timepoints (>70). However, average physical well-being was clinically low (≤18) on day 7, and average functional well-being was clinically low (≤14) on days 7–30. On the PROMIS scales, average fatigue was mild (mild range = 55–59) on days 0–30. Average pain interference was mild (mild range = 55–59) on day 14. Average physical function was mildly impaired at baseline and day 0 (mild range = 41–45), moderately impaired on days 7–30 (moderate range = 31–40), and returned to mildly impaired on day 60 (mild range = 41–45). Average ability to participate in social roles and activities was mildly impaired on days 7–30 (mild range = 41–45). All other PROMIS scale scores were in the normal range. In the week following CAR-T infusion, average FACT-G7 total scores indicated low HRQOL on days 2 and 4 (≤16).

**Figure 2:**
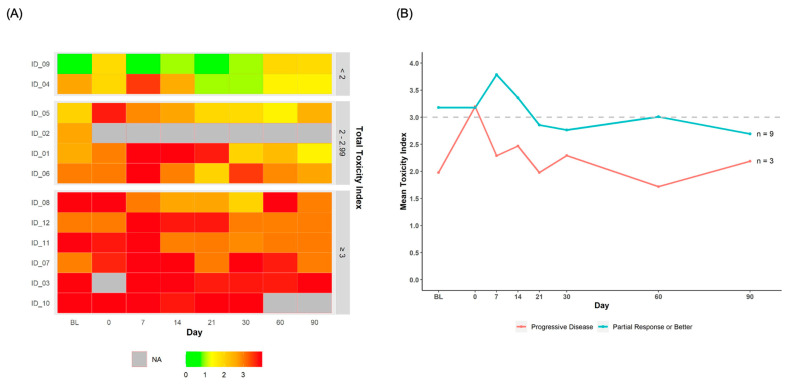
(**A**) Heatmap of toxicity index scores for each participant across time, listed from lowest to highest average within-person Toxicity Index. Abbreviations: BL, baseline; NA, not applicable/data missing. (**B**) Average toxicity index scores over time by participants’ treatment response at day 90 (partial response or better vs. progressive disease). The dashed grey line represents a Toxicity Index score of 3.0 so that scores can be easily identified as mild to moderate (<3.0) vs. severe (≥3.0). Abbreviations: BL, baseline.

Toxicity burden. The average TI score was severe (>3) on days 0, 7, and 14. Figure 2A shows a heatmap of individual TI scores across participants, listed from lowest to highest average within-person TI over time. Half of the participants (n = 6, 50%) had a within-person average TI score in the severe range (≥3).

Activity data. Average daily steps were lowest between days 0–14 (all <3000). Average sleep efficiency was in the normal range (≥85%) at all time points.

Visualizing PRO and activity data with clinical and laboratory outcomes. Figure 2B shows the average TI scores over time by participants’ treatment response at day 90. For participants with a partial response or better (n = 9, 75%), the average TI score was severe (≥3) at baseline, day 0, day 7, day 14, and day 60. For participants with progressive disease (n = 3, 25%), the average TI score was severe only on day 0.

**Figure 3:**
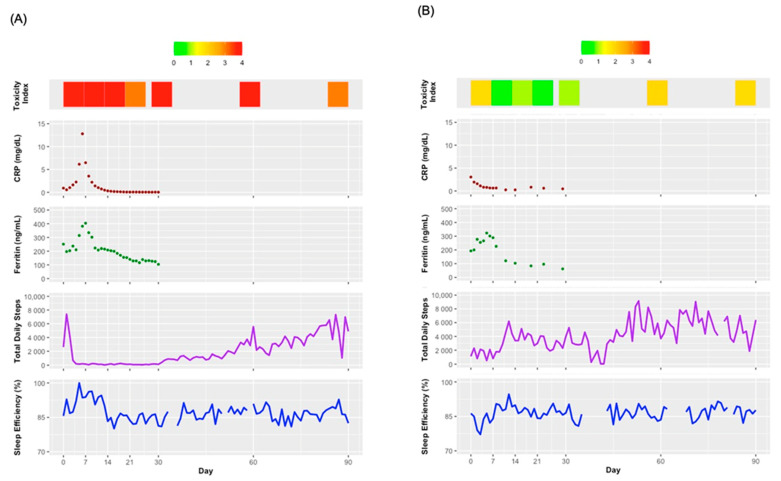
(**A**) Toxicity index scores, CRP, ferritin, total daily steps, and sleep efficiency over time for an example participant who had a partial response or better at day 90 but had a prolonged hospitalization post-CAR-T infusion. (**B**) Toxicity Index scores, CRP, ferritin, total daily steps, and sleep efficiency over time for an example participant who had progressive disease at day 90. Abbreviations: CRP, C-reactive protein; mg/dL, milligrams per deciliter; ng/mL, nanograms per milliliter.

Figure 3 shows the TI scores, laboratory values for CRP and ferritin, daily steps, and daily sleep efficiency over time for two example participants. The participant shown in Figure 3A achieved a partial response or better at day 90, but they had a complicated course with a prolonged hospitalization post-infusion. They reported at least one severe toxicity (TI ≥ 3) at most timepoints and experienced spikes in CRP and ferritin that peaked at day 7. Their daily steps declined rapidly post-infusion to <1000 steps per day through day 30, and their daily steps did not recover until approximately day 60. Their sleep efficiency increased post-infusion to almost 100% before stabilizing to around 85% after day 14. As a comparison, the participant in Figure 3B had progressive disease at day 90. They reported mild to moderate toxicities at most timepoints and experienced less extreme elevations in CRP and ferritin between days 0 and 7 relative to the participant in Figure 3A. Their daily steps were low during the week post-infusion, after which they increased fairly steadily. Their sleep efficiency decreased post-infusion and stabilized to around 85% after day 7.

## 4. Discussion

To our knowledge, this was the first study to describe longitudinal PRO and activity data collection among patients with hematologic malignancies during CAR-T, a paradigm-shifting treatment with considerable risk for severe toxicities. It was feasible and acceptable for participants to complete PRO surveys on a rigorous schedule and wear a Fitbit tracker continuously, starting pre-CAR-T infusion through 90 days post-infusion. Rates of recruitment, retention, and adherence to the procedures as well as participants’ satisfaction exceeded a priori benchmarks. Future studies should implement these procedures on a larger scale, in clinical trials as well as in standard of care, to generate valuable data that could inform clinical practice.

When collected on a larger scale, longitudinal PROs and activity data during CAR-T can be combined with routinely collected clinical data (e.g., laboratory values for ferritin, CRP) [25] to yield rich, multi-source datasets. These datasets could be used to specify risk prediction models to identify predictors of suboptimal clinical outcomes, such as severe toxicities. Given the complexity and vast amounts of data generated from these sources, machine-learning techniques may be especially useful [41]. As CAR-T is increasingly integrated into standard of care clinical practice and more ambulatory settings [42], post-infusion emergency room visits and hospital (re)admissions may become particularly important predictors of clinical outcomes as well as outcomes themselves. In turn, risk prediction models could be integrated with clinical workflows to identify patients at risk for developing CAR-T-related toxicities in real time and inform early interventions to mitigate toxicity escalation [43]. Downstream, this could improve patient outcomes.

Systems and workflows to monitor patients’ PROs and activity may also broaden access to care. Currently, CAR-T is limited to highly specialized cancer centers, due in part to the potential for severe toxicities that necessitates close observation post-infusion [44], and patients must sometimes stay within close proximity of the treating center for up to one month. This has created disparities in access to care based on physical proximity and/or means to travel for extended periods of time [45]. However, with the integration of systematic monitoring and validated risk prediction models into clinical care, it may be possible for clinicians in community clinics to treat and monitor patients in collaboration with off-site clinicians in specialized cancer centers. Similar models in cancer care have already been successfully implemented. For example, the collaboration between Project ECHO (Extension for Community Healthcare Outcomes) and MD Anderson Cancer Center connects clinicians in rural and underserved settings with educational and mentoring resources to empower community clinicians and improve their ability to manage patients with complex presentations [46,47]. Future work should explore the application of similar models to scale up the delivery of CAR-T in community settings.

Visually, preliminary data patterns suggested that patients with a partial treatment response or better had a higher toxicity burden than patients with progressive disease. These preliminary data patterns are largely hypothesis generating and require replication in larger samples with appropriate power to test for statistically significant differences between groups. Nonetheless, these observations complement an emerging body of research evaluating the link between the severity of clinician-reported toxicities and patients’ clinical outcomes. For example, Brammer and colleagues found that patients with lymphoma who developed moderate (grade 2) CRS post-CAR-T infusion had better disease response than patients with mild (grade 1) or severe (grade 3) CRS [48]. Future studies should combine clinician-reported toxicities with other data sources, such as PROs, to identify early risk factors for suboptimal clinical outcomes [49]. In turn, PRO data may be used to help researchers develop novel strategies to augment CAR-T therapies, as needed.

Limitations. This study had a small sample size and was underpowered to explore the statistical significance of changes in PROs and activity over time, the relationships of PROs and activity data with clinical outcomes, or differences in outcomes based on specific diagnoses or CAR-T regimens. In addition, we did not consider all possible data collected by the Fitbit trackers (e.g., heart rate), nor did we use wearable devices capable of measuring other biometric variables (e.g., oxygenation, temperature). Future large-scale studies should investigate these relationships and additional biometric endpoints. Participants were mostly White and non-Hispanic, which may limit the generalizability of findings to more racially and ethnically diverse populations. In addition, this study did not include an assessment of digital health literacy prior to enrolling participants, which future studies should consider.

## 5. Conclusions

This study showed that it is feasible and acceptable to collect longitudinal PRO data on a rigorous schedule and activity data via wearable devices among patients with hematologic malignancies receiving CAR-T. Findings suggest that large-scale data collection efforts will be successful. Future studies can use these procedures to generate rich, multi-source datasets to specify risk prediction models via machine learning that will ultimately help identify risk factors for severe CAR-T-related toxicities, inform early toxicity management interventions, improve patient outcomes, and broaden access to care.

## Figures and Tables

**Table 1:** Schedule and content of PRO surveys.

Measure	Construct	Baseline	Day 0	Days 1–6	Day 7	Day 14	Day 21	Day 30	Day 60	Day 90
Demographics survey	Demographics	X								
CCI	Comorbidities	X								
FACT-G	HRQOL	X	X		X	X	X	X	X	X
PROMIS-29 + 2 Profile v2.1	HRQOL	X	X		X	X	X	X	X	X
FACT-G7	HRQOL			X						
PRO-CTCAE items	Toxicity burden	X	X		X	X	X	X	X	X
Study-specific survey	Acceptability							X		

Abbreviations: CCI, Charlson Comorbidity Index; FACT-G, Functional Assessment of Cancer Therapy-General; HRQOL, health-related quality of life; PRO-CTCAE, Patient-Reported Outcomes Version of the Common Terminology Criteria for Adverse Events; PROMIS, Patient-Reported Outcomes Measurement Information System.

**Table 2:** Participants’ demographic and clinical characteristics (N = 12).

Variable	Statistic
Age, years; M (SD) range	66 (7) 53–77
Male gender; n (%)	6 (50)
Race; n (%)	
White	10 (83)
Black/African American	2 (17)
Non-Hispanic; n (%)	11 (92)
Highest education completed; n (%)	
Partial college or specialized training	7 (58)
College or university	4 (33)
Graduate degree	1 (8)
Annual household income; n (%)	
USD20,000–USD39,999	2 (17)
USD40,000–USD59,999	1 (8)
USD60,000–USD100,000	3 (25)
>USD100,000	3 (25)
Prefer not to report	3 (25)
Cancer diagnosis; n (%)	
Multiple myeloma	7 (58)
Mantle cell lymphoma	3 (25)
Chronic lymphoid leukemia	1 (8)
Diffuse large B cell lymphoma	1 (8)
Hospital length of stay, days; M (SD) range	10 (7) 6–32
Charlson Comorbidity Index; M (SD) range	3 (1) 2–6

Note: Percentages may not sum to 100 due to rounding. The following variables had missing data: ethnicity (n = 1 missing), Charlson Comorbidity Index (n = 1 missing). Abbreviations: M, mean; n, frequency; SD, standard deviation.

**Table 3:** Descriptive statistics of HRQOL, toxicity burden, and activity data across time (N = 12).

Measure/Scale	Baseline	Day 0	Day 7	Day 14	Day 21	Day 30	Day 60	Day 90
FACT-G; M (SD)								
Total HRQOL	81 (17)	77 (19)	75 (18)	73 (18)	75 (21)	79 (19)	85 (19)	84 (19)
Physical well-being	21 (5)	20 (6)	18 (7)	19 (8)	19 (7)	21 (5)	23 (5)	23 (5)
Social well-being	23 (5)	23 (5)	23 (5)	22 (5)	22 (5)	23 (5)	23 (5)	23 (6)
Emotional well-being	19 (4)	20 (3)	21 (2)	20 (5)	20 (4)	20 (3)	21 (3)	20 (4)
Functional well-being	17 (7)	15 (7)	13 (7)	13 (5)	14 (7)	14 (7)	18 (7)	18 (8)
PROMIS-29 + 2 Profile v2.1; M (SD)								
Depression	48 (7)	47 (7)	46 (6)	48 (9)	48 (9)	47 (8)	47 (7)	45 (7)
Anxiety	46 (7)	46 (7)	45 (6)	47 (10)	47 (8)	48 (9)	46 (8)	45 (7)
Fatigue	52 (11)	56 (12)	57 (13)	58 (13)	56 (11)	55 (12)	50 (10)	47 (10)
Pain interference	51 (9)	52 (12)	54 (10)	55 (8)	53 (11)	52 (12)	50 (9)	49 (9)
Physical function	43 (8)	42 (7)	38 (9)	37 (11)	39 (10)	40 (9)	44 (8)	46 (8)
Sleep disturbance	52 (10)	54 (9)	53 (9)	50 (11)	48 (8)	48 (11)	48 (9)	47 (9)
Social roles and activities	51 (10)	48 (11)	45 (12)	44 (10)	45 (10)	44 (9)	52 (10)	52 (10)
Cognitive function	55 (7)	53 (9)	52 (9)	50 (7)	50 (7)	53 (7)	55 (8)	56 (6)
Global pain	3 (3)	3 (3)	4 (3)	3 (3)	3 (3)	3 (3)	2 (3)	3 (2)
Toxicity index; M (SD)	2.9 (1.1)	3.2 (0.8)	3.4 (1.2)	3.1 (0.9)	2.6 (1.3)	2.6 (1.1)	2.8 (0.9)	2.6 (0.8)
Daily steps; M (SD) ^a^	-	2155 (1425)	1923 (947)	2585 (1817)	3171 (2273)	3236 (2030)	4575 (2083)	5312 (1834)
Sleep efficiency; M (SD) ^a^	-	86% (3)	86% (4)	88% (4)	86% (2)	86% (3)	87% (1)	87% (1)
**Measure**	**Day 1**	**Day 2**	**Day 3**	**Day 4**	**Day 5**	**Day 6**		
FACT-G7 total HRQOL; M (SD)	17 (8)	16 (5)	17 (4)	15 (5)	18 (5)	17 (6)		

Note: ^a^ Daily values were averaged across the preceding timeframe (e.g., day 14 is the average of days 8–14). There are no baseline values for daily steps or sleep efficiency because participants were instructed to begin wearing the Fitbit tracker on day 0. Abbreviations: FACT-G, Functional Assessment of Cancer Therapy-General; PROMIS, Patient-Reported Outcome Measurement Information System; HRQOL, health-related quality of life; M, mean; SD, standard deviation.

## Data Availability

The data that support these findings are available from the corresponding author upon reasonable request.
